# P14 Knowledge, attitude and practices on antibiotic usage and resistance among people attending primary healthcare in Rwanda

**DOI:** 10.1093/jacamr/dlae136.018

**Published:** 2024-08-23

**Authors:** Jerome Ndayisenga, Obed Tuyishimire, Olivier Sibomana, Philemon Kwizera, Hosee Niyompano, François Hakizayezu, Margaret I Fitch

**Affiliations:** Research, Innovation and Data Science Division, Rwanda Biomedical Centre (RBC), Kigali, Rwanda; Department of Biomedical Laboratory Sciences, School of Health Sciences, College of Medicine and Health Sciences, University of Rwanda, Rwanda; Research for Development, Kigali, Rwanda; Department of General Medicine and Surgery, School of Medicine and Pharmacy, College of Medicine and Health Sciences, University of Rwanda, Butare, Rwanda; Department of Human Nutrition and Dietetics, School of Public Health, College of Medicine and Health Sciences, University of Rwanda, Kigali, Rwanda; National Reference Laboratory, Rwanda Biomedical Centre, Kigali, Rwanda; National Reference Laboratory, Rwanda Biomedical Centre, Kigali, Rwanda; Bloomberg Faculty of Nursing, University of Toronto, Toronto, Canada

## Abstract

**Background:**

Antimicrobial resistance (AMR) poses a global threat to public health, with Sub-Sahara African countries facing a substantial burden. Our study aimed to assess knowledge, attitude and awareness of antibiotic usage and resistance among people attending primary healthcare facilities in Rwanda.

**Methods:**

The study was descriptive cross-sectional and proportionate stratified sampling was used to recruit 246 individuals who attended health centres in Kigali during October 2023. The study assessed the level of knowledge on antibiotic usage, knowledge on antimicrobial resistance, attitudes toward antibiotic accessibility and practices regarding the patient-prescriber relationship and infection prevention. The levels were calculated as proportions of correct answers and were grouped as poor (below 40%), moderate (from 40 and less than 70%) and high, good or positive (70% and higher). χ^2^ test was used find significance of associations between levels and age, gender and education.

**Results:**

Most participants (87.40%) correctly identified Amoxicillin as an antibiotic while 57.72% wrongly identified Paracetamol as an antibiotic. Among participants, 3.2% had high knowledge on antibiotic usage, 20.7% had high knowledge on antimicrobial resistance, 32.9% had a positive attitude toward antibiotic accessibility and 39.4% had good practices regarding the patient-prescriber relationship and infection control. Male participants had significantly higher levels of positive attitude (*P*=0.003) and knowledge on antimicrobial resistance (*P*=0.047). Individuals with university education had significantly higher levels of positive attitude (*P*=0.007).

**Conclusions:**

Limited levels of knowledge, attitude and practices on antibiotic usage and resistance were found, with women having lower levels in multiple aspects. Strategies to promote rational use of antibiotics ought to address social inequities.

